# Supplementary material for: Site-specific fabrication of a melanin-like pigment through spatially confined progressive assembly on an initiator-loaded template
Source: Nat Commun. 2023 Jun 10;14:3432. doi: 10.1038/s41467-023-38622-2 (PMC10257687; doi:10.1038/s41467-023-38622-2)
Supplement: Supplementary file 1 — Supplementary information [file 41467_2023_38622_MOESM1_ESM.pdf]

# Supplementary Information

**Site-specific fabrication of a melanin-like pigment through spatially confined progressive assembly on an initiator-loaded template**

*Haejin Jeong, Jisoo Lee, Seunghwi Kim, Haeram Moon, and Seonki Hong**

Prof. Dr. S. Hong, H. Jeong, J. Lee, H. Moon, S. Kim
Department of Physics and Chemistry, DGIST, Daegu, 42988, Republic of Korea
*Correspondence to: seonkihong@dgist.ac.kr

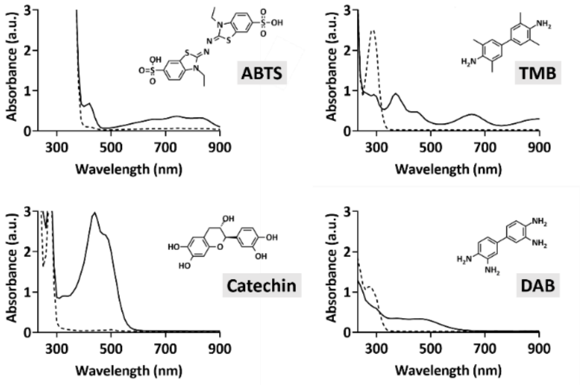


**Supplementary Fig. 1**. UV–vis-NIR spectrum of four class 1 precursors showing defined peaks corresponding to water-soluble chromophores after treatment with HRP/$H_2O_2$ (HRP: 10 U/mL, $H_2O_2$ 20 mM) in solution for 60 min at 25 °C (dotted line: intrinsic spectrum of precursor before oxidation, solid line: spectrum after HRP/$H_2O_2$ oxidation). ABTS: 2,2'-Azino-bis(3-ethylbenzothiazoline-6-sulfonic acid), TMB: 3,3',5,5'-Tetramethylbenzidine, DAB: 3,3-diaminobenzidine, a.u.: arbitrary units.

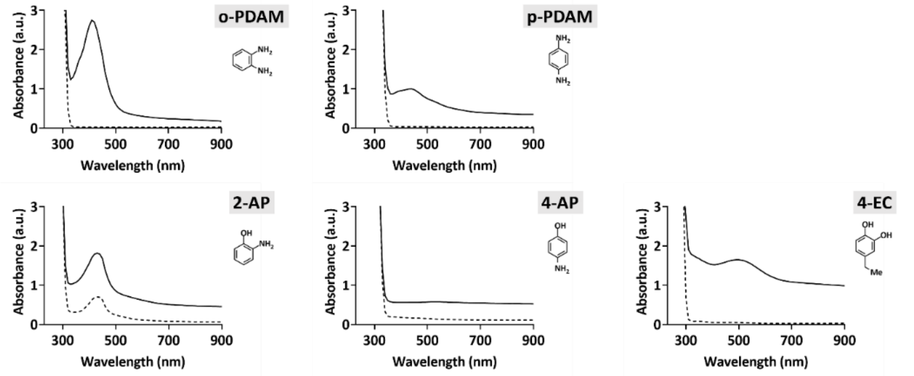


**Supplementary Fig. 2**. UV–vis-NIR spectrum of five class 2 precursors resulting in nano-to-micro-sized particulate formation after treatment with HRP/$H_2O_2$ (HRP: 10 U/mL, $H_2O_2$ 20 mM) in solution for 60 min at 25 °C (dotted line: intrinsic spectrum of precursor before oxidation, solid line: spectrum after HRP/$H_2O_2$ oxidation). PDAM: phenylenediamine, AP: aminophenol, EC: ethylcatechol, a.u.: arbitrary units.

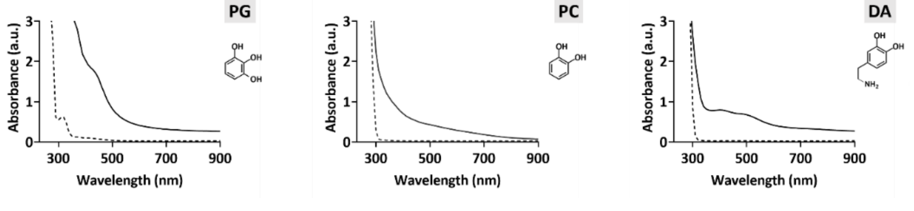


**Supplementary Fig. 3**. UV–vis-NIR spectrum of three class 3 precursors showing melanin-like pigmentation behavior after treatment with HRP/$H_2O_2$ (HRP: 10 U/mL, $H_2O_2$ 20 mM) in solution for 60 min at 25 °C (dotted line: intrinsic spectrum of precursor before oxidation, solid line: spectrum after HRP/$H_2O_2$ oxidation). PG: pyrogallol, PC: pyrocatechol, DA: dopamine, a.u.: arbitrary units.

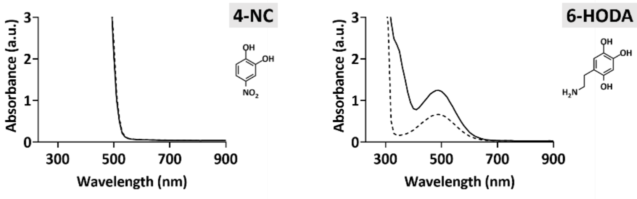


**Supplementary Fig. 4**. UV–vis-NIR spectrum of two class 4 precursors having intrinsic background signals before treatment with HRP/$H_2O_2$ (HRP: 10 U/mL, $H_2O_2$ 20 mM) in solution for 60 min at 25 °C (dotted line: intrinsic spectrum of precursor before oxidation, solid line: spectrum after HRP/$H_2O_2$ oxidation). NC: nitrocatechol, 6-HODA: 6-hydroxydopamine, a.u.: arbitrary units.

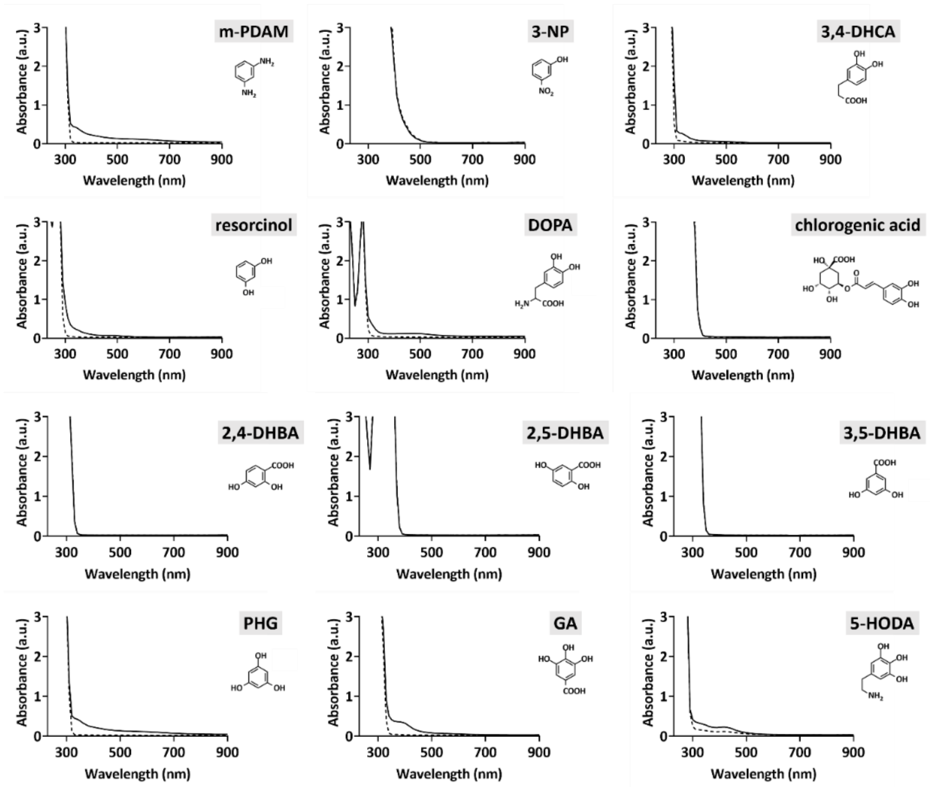


**Supplementary Fig. 5**. UV–vis-NIR spectrum of class 5 precursors having minimum response after treatment with HRP/$H_2O_2$ (HRP: 10 U/mL, $H_2O_2$ 20 mM) in solution for 60 min at 25 °C (dotted line: intrinsic spectrum of precursor before oxidation, solid line: spectrum after HRP/$H_2O_2$ oxidation). NP: nitrophenol, DHCA: dihydrocaffeic acid, DOPA: dihydroxyphenylalanine, DHBA: dihydroxybenzoic acid, PHG: phloroglucinol, GA: gallic acid, 5-HODA: 5-hydroxydopamine, a.u.: arbitrary units.

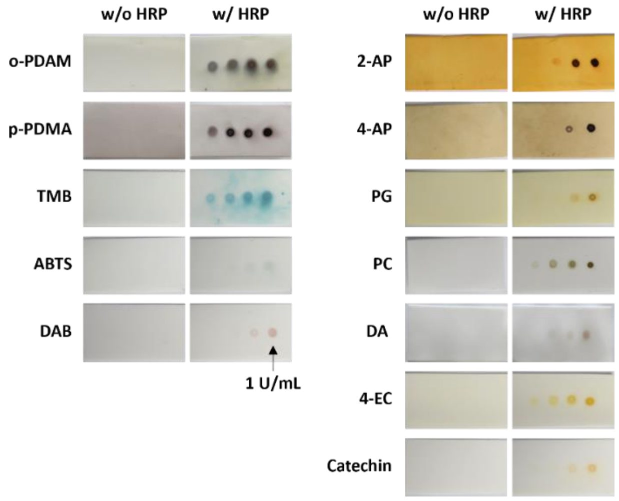


**Supplementary Fig. 6**. Precursor-dependent behavior of surface-initiated pigment generation through the PAINT approach. The membrane without HRP was simply immersed in the precursor solution, showing the intrinsic color of unreacted precursors. The membrane with HRP contained four 1 μL-sized spots of 0.001, 0.01, 0.1 and 1 U/mL HRP, which were visualized by in situ generated and attached pigments.

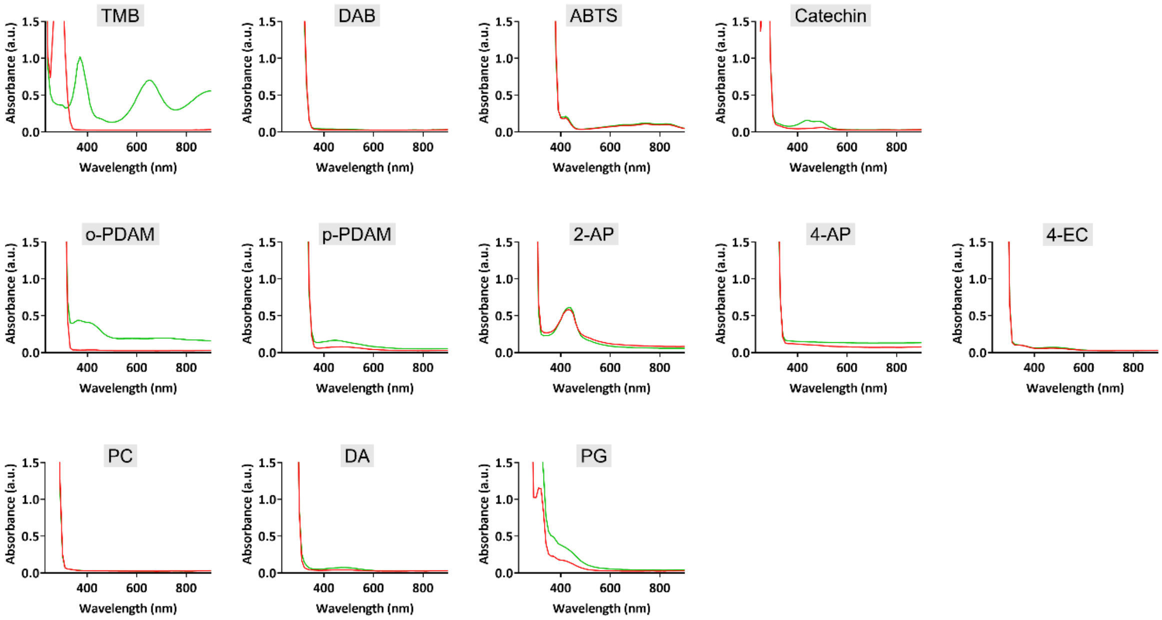


**Supplementary Fig. 7**. UV–visible spectra of diffused pigment in solution during surface-initiated pigmentation of various precursors (red: incubation w/o HRP-spotted membrane, green: incubation with HRP-spotted membrane). a.u.: arbitrary units.

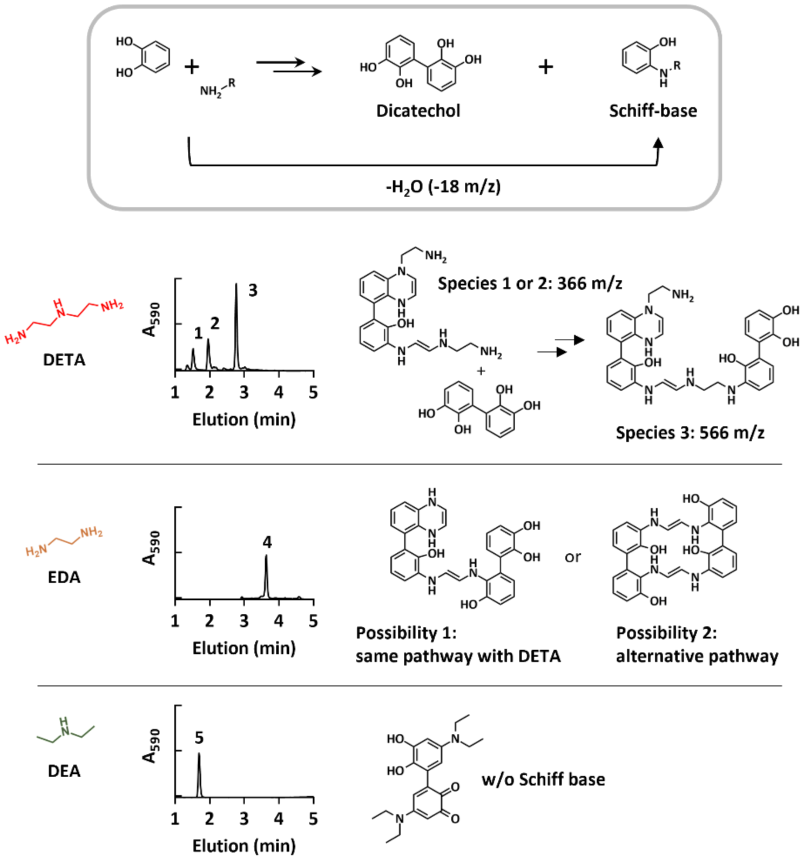


**Supplementary Fig. 8**. Plausible structures of water-soluble intermediates identified from the PC-based pigmentation with three different amine additives through HPLC-MS analysis.

| R-NH2 | Species | Elution time | m/z | Catechol | Primary amine | Schiff-base |
|---|---|---|---|---|---|---|
| | 1 | 1.5 min | 366 | 2 | 4 | 3 |
| DETA | 2 | 2.0 min | 366 | 2 | 4 | 3 |
| | 3 | 2.7 min | 566 | 4 | 4 | 4 |
| EDA | 4 | 3.6 min | 480 | 4 | 4 | 4 |
| DEA | 5 | 1.7 min | 358 | 2 | 2 | - |

**Supplementary Table 1**. HPLC–MS analysis of water-soluble intermediates identified from the PC-based pigmentation with three different amine additives.

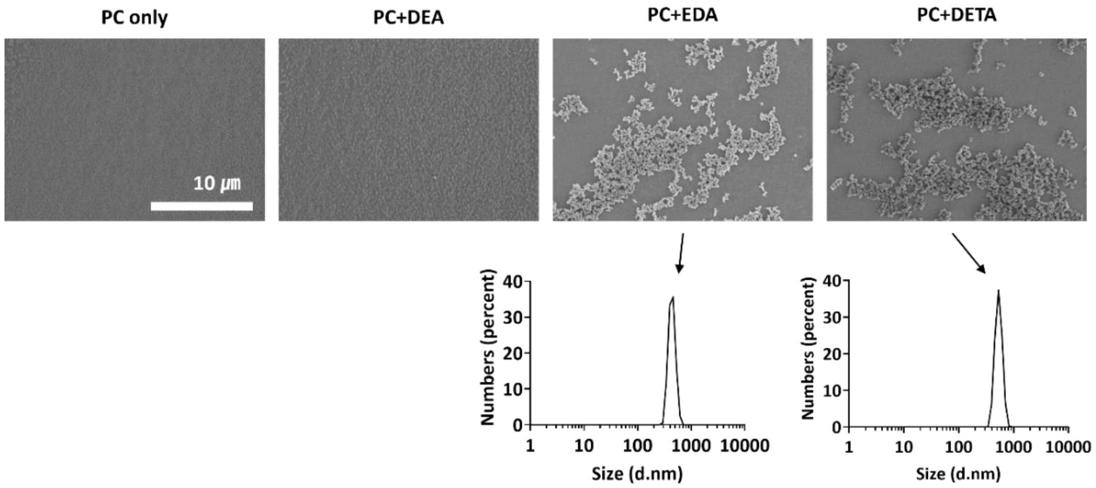


**Supplementary Fig. 9**. Aggregates formed during the solution-phase reaction of PC and amine additives with HRP. The aggregates were thoroughly filtered out before HPLC-MS analysis.

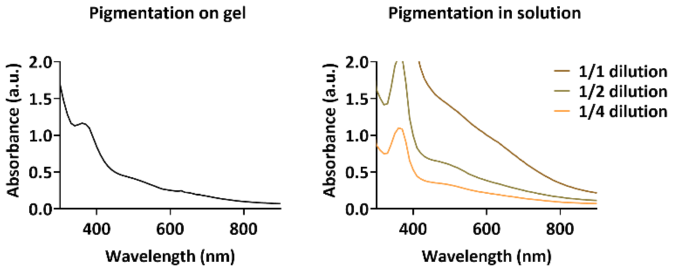


**Supplementary Fig. 10**. Comparison of solution-synthesized pigment and the pigment fabricated on a gel by PAINT approach. a.u.: arbitrary units.

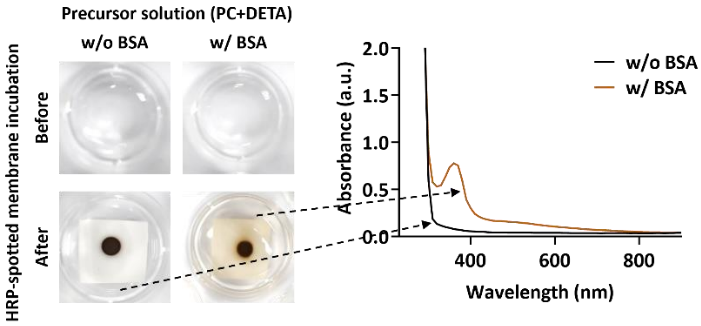


**Supplementary Fig. 11**. UV–visible spectrum of pigment that had diffused into the solution during surface-initiated pigment fabrication by the PAINT approach in the presence of BSA in the precursor solution. a.u.: arbitrary units.

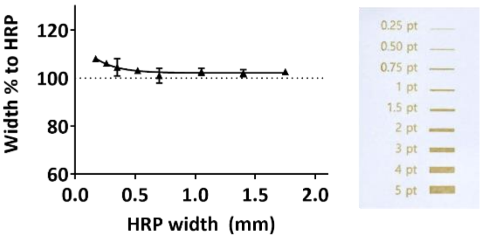


**Supplementary Fig. 12**. Width expansion% of PC-based pigment from each inkjet-printed HRP pattern with different widths ranging from 0.2 to 1.75 mm.

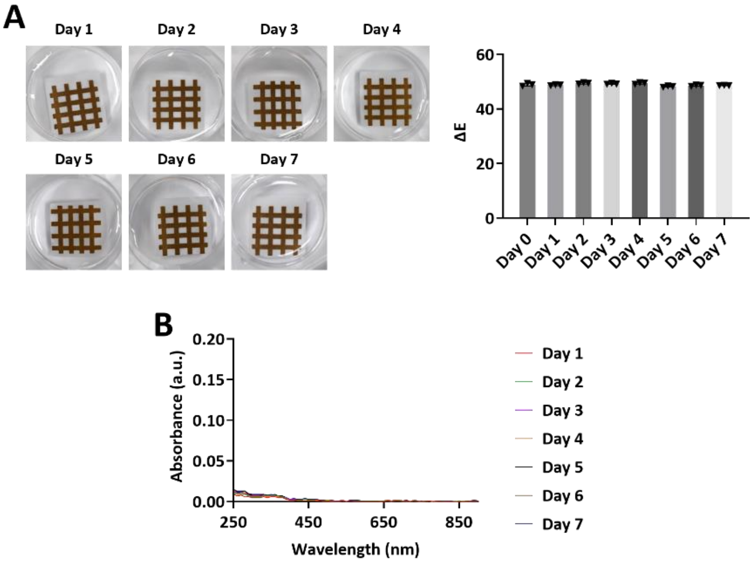


**Supplementary Fig. 13**. Intensity monitoring of PC/DETA pigment (A) and the detection of leachable products formed (B) during the incubation of printed PC/DETA pigment maintained in 1×PBS (pH 7.4) at 37 °C for 7 days. a.u.: arbitrary units.

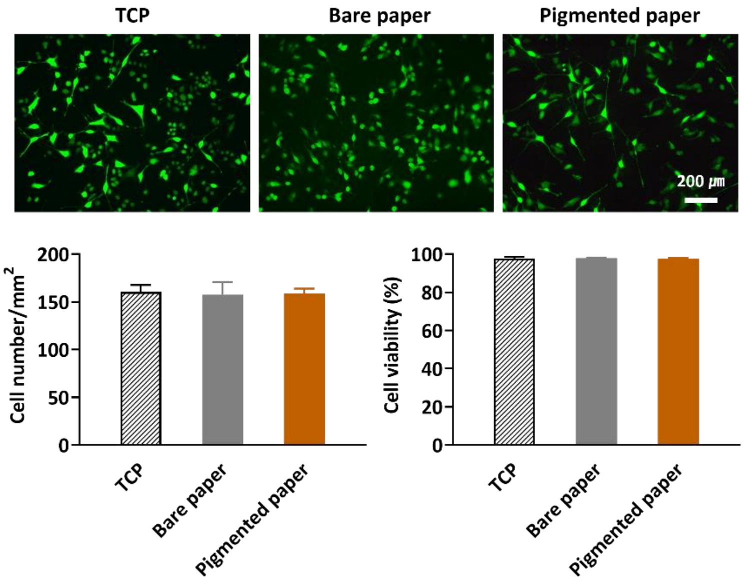


**Supplementary Fig. 14**. Comparison of cell adhesion and viability of conventional tissue culture plate (TCP), bare A4 paper and pigmented A4 paper.

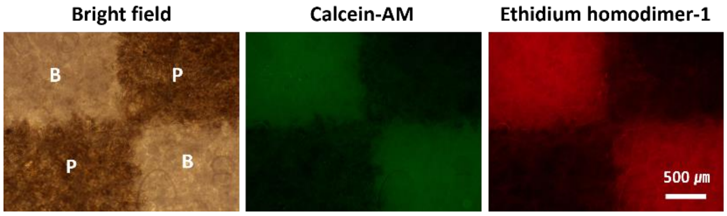


**Supplementary Fig. 15**. Attenuation of background fluorescence from the pigmented region of A4 paper.

| | Chemical | Purity | Vendor |
|---|---|---|---|
| 1 | p-Phenylenediamiine (p-PDAM) | 98% | Sigma Aldrich |
| 2 | o-Phenylenediamiine (o-PDAM) | 98% | Sigma Aldrich |
| 3 | 3-Nitrophenol (3-NP) | 99.50% | Sigma Aldrich |
| 4 | 2-Aminophenol (2-AP) | 99.90% | Sigma Aldrich |
| 5 | 4-Aminophenol (4-AP) | 100% | Sigma Aldrich |
| 6 | 4-Ethylcatechol (4-EC) | 95% | Sigma Aldrich |
| 7 | 4-Nitrocatechol (4-NC) | 99.10% | Sigma Aldrich |
| 8 | 3,4-Dihydroxy-L-phenylalanine (DOPA) | 100% | Sigma Aldrich |
| 9 | 2,4-Dihydroxybenzoic acid (2,4-DHBA) | 99.90% | Sigma Aldrich |
| 10 | Pyrocatechol (PC) | 99% | Sigma Aldrich |
| 11 | Dopamine hydrochloride (DA) | 98% | Sigma Aldrich |
| 12 | 3,4-Dihydroxyhydrocinnamic acid (3,4-DHCA) | 98% | Sigma Aldrich |
| 13 | 3,5-Dihydroxybenzoic acid (3,5-DHBA) | 99.90% | Sigma Aldrich |
| 14 | Chlorogenic acid | 98% | TCI |
| 15 | 2,5-Dihydroxybenzoic acid (2,5-DHBA) | 98% | Sigma Aldrich |
| 16 | Resorcinol | 99% | Sigma Aldrich |
| 17 | Phloroglucinol (PHG) | 99.70% | Sigma Aldrich |
| 18 | Gallic acid (GA) | 99.20% | Sigma Aldrich |
| 19 | 5-Hydroxydopamine hydrochloride (5-HDA) | 98% | Sigma Aldrich |
| 20 | 6-Hydroxydopamine hydrochloride (6-HDA) | 100% | Sigma Aldrich |
| 21 | Catechin hydrate | 99% | Sigma Aldrich |
| 22 | Pyrogallol (PG) | 100% | Sigma Aldrich |
| 23 | 3,3',5,5'-Tetramethylbenzidine (TMB) | 100% | Sigma Aldrich |
| 24 | 2,2'-Azino-bis(3-ethylbenzothiazoline-6-sulphonic acid (ABTS) | 100% | Sigma Aldrich |
| 25 | 3, 3-Diaminobenzidine (DAB) | 97.50% | Sigma Aldrich |
| 26 | Hydrogen peroxide ($H_2O_2$) | 30wt. % in $H_2O$ | Sigma Aldrich |
| 27 | Diethylenetriamine (DETA) | 99.90% | Sigma Aldrich |
| 28 | Diethylamine (DEA) | 99.90% | Sigma Aldrich |
| 29 | Ethylenediamine (EDA) | 99.92% | Sigma Aldrich |
| 30 | Trifluoroacetic acid (TFA) | 99.00% | Sigma Aldrich |
| 31 | Acetonitrile (ACN) | 100% | Sigma Aldrich |

**Supplementary Table 2**. Purities of purchased materials.
