# Supplementary material for: Site-specific fabrication of a melanin-like pigment through spatially confined progressive assembly on an initiator-loaded template
Source: Nat Commun. 2023 Jun 10;14:3432. doi: 10.1038/s41467-023-38622-2 (PMC10257687; doi:10.1038/s41467-023-38622-2)
Supplement: Supplementary file 3 — Reporting Summary [file 41467_2023_38622_MOESM3_ESM.pdf]

nature portfolio

Corresponding author(s): Seonki Hong

Last updated by author(s): Apr 7, 2023

Reporting Summary

Nature Portfolio wishes to improve the reproducibility of the work that we publish. This form provides structure for consistency and transparency in reporting. For further information on Nature Portfolio policies, see our Editorial Policies and the Editorial Policy Checklist.

Statistics

For all statistical analyses, confirm that the following items are present in the figure legend, table legend, main text, or Methods section.

n/a	Confirmed	
☐	☒	The exact sample size (*n*) for each experimental group/condition, given as a discrete number and unit of measurement
☐	☒	A statement on whether measurements were taken from distinct samples or whether the same sample was measured repeatedly
☒	☐	The statistical test(s) used AND whether they are one- or two-sided<br>*Only common tests should be described solely by name; describe more complex techniques in the Methods section.*
☒	☐	A description of all covariates tested
☒	☐	A description of any assumptions or corrections, such as tests of normality and adjustment for multiple comparisons
☐	☒	A full description of the statistical parameters including central tendency (e.g. means) or other basic estimates (e.g. regression coefficient) AND variation (e.g. standard deviation) or associated estimates of uncertainty (e.g. confidence intervals)
☒	☐	For null hypothesis testing, the test statistic (e.g. *F*, *t*, *r*) with confidence intervals, effect sizes, degrees of freedom and *P* value noted<br>*Give P values as exact values whenever suitable.*
☒	☐	For Bayesian analysis, information on the choice of priors and Markov chain Monte Carlo settings
☒	☐	For hierarchical and complex designs, identification of the appropriate level for tests and full reporting of outcomes
☒	☐	Estimates of effect sizes (e.g. Cohen's *d*, Pearson's *r*), indicating how they were calculated

Our web collection on statistics for biologists contains articles on many of the points above.

Software and code

Policy information about availability of computer code

Data collection	UV–vis spectrophotometer data were collected using Bio Tek Gen5 software (v 3.04.17). The fluorescence microscope images were collected using Dixi eXcope software (v x64). HPLC-MS data were collected using Agilent MassHunter LCMS Acquisition Console (v B.05.00) and Qualitative analysis (v B.06.00). Scanning Electron Microsope (SEM) images were collected using Hitachi S-4800 (operation v 3.21 and evacuation v 1.8). Samsung SL-J1560 Series PCL-3 (v 25.1) was used for HRP printing.
Data analysis	ImageJ (v 1.53k) was used for image analysis. ChemBioDraw (v 12.0) was used for chemical formula. Graphpad Prism (v 9) was used for data presentation.

For manuscripts utilizing custom algorithms or software that are central to the research but not yet described in published literature, software must be made available to editors and reviewers. We strongly encourage code deposition in a community repository (e.g. GitHub). See the Nature Portfolio guidelines for submitting code & software for further information.

Data

Policy information about availability of data

All manuscripts must include a data availability statement. This statement should provide the following information, where applicable:

- Accession codes, unique identifiers, or web links for publicly available datasets
- A description of any restrictions on data availability
- For clinical datasets or third party data, please ensure that the statement adheres to our policy

All data needed to support the conclusions in the paper are present in the paper and/or the Supplementary Information. Source data are provided with this paper.

Human research participants

Policy information about studies involving human research participants and Sex and Gender in Research.

Reporting on sex and gender	N/A
Population characteristics	N/A
Recruitment	N/A
Ethics oversight	N/A

Note that full information on the approval of the study protocol must also be provided in the manuscript.

Field-specific reporting

Please select the one below that is the best fit for your research. If you are not sure, read the appropriate sections before making your selection.

☒ Life sciences ☐ Behavioural & social sciences ☐ Ecological, evolutionary & environmental sciences

For a reference copy of the document with all sections, see nature.com/documents/nr-reporting-summary-flat.pdf

Life sciences study design

All studies must disclose on these points even when the disclosure is negative.

Sample size	Sample size was chosen to ensure reproducibility of the experiments and to support meaningful conclusions. The sample size of each experiment is provided in figure legends.
Data exclusions	Only data for experiments that failed due to technical issues were excluded.
Replication	All experiments were replicated multiple times with reproducible results and indicated in figure legends.
Randomization	We don't have any in vivo and human studies to consider randomization.
Blinding	We don't have any in vivo and human studies to consider blinding.

Reporting for specific materials, systems and methods

We require information from authors about some types of materials, experimental systems and methods used in many studies. Here, indicate whether each material, system or method listed is relevant to your study. If you are not sure if a list item applies to your research, read the appropriate section before selecting a response.

March 2021

Materials & experimental systems

n/a	Involved in the study
☒	☐ Antibodies
☐	☒ Eukaryotic cell lines
☒	☐ Palaeontology and archaeology
☒	☐ Animals and other organisms
☒	☐ Clinical data
☒	☐ Dual use research of concern

Methods

n/a	Involved in the study
☒	☐ ChIP-seq
☒	☐ Flow cytometry
☒	☐ MRI-based neuroimaging

Eukaryotic cell lines

Policy information about cell lines and Sex and Gender in Research

Cell line source(s)	NIH3T3 cell line and HeLa cell line purchased from ATCC.
Authentication	The cell lines were certificated by the manufacturers (ATCC).
Mycoplasma contamination	We confirmed that all cell lines tested negative for mycoplasma contamination.
Commonly misidentified lines (See ICLAC register)	No commonly misidentified cell lines were used.
